# Sagittal plane assessment of spino-pelvic complex in a Central European population with adolescent idiopathic scoliosis: a case control study

**DOI:** 10.1186/s13013-018-0156-0

**Published:** 2018-06-14

**Authors:** Máté Burkus, Ádám Tibor Schlégl, Ian O’Sullivan, István Márkus, Csaba Vermes, Miklós Tunyogi-Csapó

**Affiliations:** 1 Department of Orthopedics, Medical School, University of Pécs, Akác st. 1, Pécs, H-7623 Hungary; 2 Department of Traumatology and Hand Surgery, Petz Aladár County Teaching Hospital, Vasvári Pál st. 2-4, Győr, H-9023 Hungary

**Keywords:** EOS 2D/3D, Sagittal alignment, Spino-pelvic parameters, Adolescent idiopathic scoliosis

## Abstract

**Background:**

Scoliosis is a complex three-dimensional deformity. While the frontal profile is well understood, increasing attention has turned to balance in the sagittal plane. The present study evaluated changes in sagittal spino-pelvic parameters in a large Hungarian population with adolescent idiopathic scoliosis.

**Methods:**

EOS 2D/3D images of 458 scoliotic and 69 control cases were analyzed. After performing 3D reconstructions, the sagittal parameters were assessed as a whole and by curve type using independent sample *t* test and linear regression analysis.

**Results:**

Patients with scoliosis had significantly decreased thoracic kyphosis (*p* < 0.001) with values T1–T12, 34.1 ± 17.1^o^ vs. 43.4 ± 12.7^o^ in control; T4–T12, 27.1 ± 18.8^o^ vs. 37.7 ± 15.1^o^ in control; and T5–T12, 24.9 ± 15.8^o^ vs. 32.9 ± 15.0^o^ in control. Changes in thoracic kyphosis correlated with magnitude of the Cobb angle (*p* < 0.001). No significant change was found in lumbar lordosis and the pelvic parameters. After substratification according to the Lenke classification and individually evaluating subgroups, results were similar with a significant decrease in only the thoracic kyphosis. A strong correlation was seen between sacral slope, pelvic incidence, and lumbar lordosis, and between pelvic version and thoracic kyphosis in control and scoliotic groups, whereas pelvic incidence was also seen to be correlated with thoracic kyphosis in scoliosis patients.

**Conclusion:**

Adolescent idiopathic scoliosis patients showed a significant decrease in thoracic kyphosis, and the magnitude of the decrease was directly related to the Cobb angle. Changes in pelvic incidence were minimal but were also significantly correlated with thoracic changes. Changes were similar though not identical to those seen in other Caucasian studies and differed from those in other ethnicities. Scoliotic curves and their effect on pelvic balance must still be regarded as individual to each patient, necessitating individual assessment, although changes perhaps can be predicted by patient ethnicity.

## Background

Scoliosis is a rotational deformity of the curvature of the spinal column. Although deformity is most marked in the frontal plane, growing evidence indicates that a detailed sagittal evaluation is necessary in addition to anteroposterior imaging for optimizing treatment planning [1–3].

The sagittal spino-pelvic parameters have been assessed in numerous publications with normal, disease-free populations [4–8] and in spine deformities, including scoliosis [9–11]*.* While many of these studies found alterations in sagittal alignment in scoliotic patients [9, 10, 12], a notable number reported no significant difference [13].

The dynamic relationship between the sagittal position of the pelvis and the spine is also evident during imaging in spine deformities such as spondylolisthesis and intervertebral disc abnormalities [6, 14, 15]. Changes are seen during growth, too, with all parameters evolving and developing throughout childhood and puberty until their attainment of mature adult values [16–18].

Despite growing agreement on the importance of sagittal alignment in scoliosis treatment, uncertainty exists in the literature about their values in health and in disease. Possible contributing factors are the limitations associated with single-plane image assessment modalities, in addition to inter-individual and even possible inter-ethnic variability [19]. In recent years, studies using the EOS 2D/3D scanner have gained popularity, as the scanner allows improved characterization of complex deformities in three dimensions. The EOS 2D/3D scanner captures standing images with minimal vertical distortion and in combination with its reconstruction software has contributed to our understanding of the biomechanical and anatomical parameters of the spine, pelvis, and lower extremities [18, 20–23].

The current study aims to assess and present data on the sagittal position of the spine and pelvis in a large sample of Central European adolescents and young adults with adolescent idiopathic scoliosis using high accuracy and low radiation EOS imaging and evaluate the relationships within the spino-pelvic complex.

## Methods

Our clinic’s radiological records were retrospectively examined for the period from 2007 to 2012, and EOS 2D/3D images for 511 AIS patients, defined as Cobb angle > 10°, were found. Patients with any other spinal deformity or those with previous spinal or lower extremity surgical intervention were excluded. Finally, 458 patients (82 male, 376 female) were available, with mean age 16.8 ± 4.7 years, range 12–26 years.

For control, 69 individuals (28 male, 41 female) free from any spinal deformity were randomly collected from our database. EOS 2D/3D scans had been indicated in these individuals due to suspected scoliosis, though this was not found to be present, or for joint pain, which was later found to lack any bone involvement. The mean age was 17.1 ± 4.4 years, range 12–26 years.

All patients or their parents/guardians gave written consent at the time of imaging for future use in clinical research. According to Hungarian regulations for retrospective analysis, further ethical permission was not required.

**Fig. 1:**
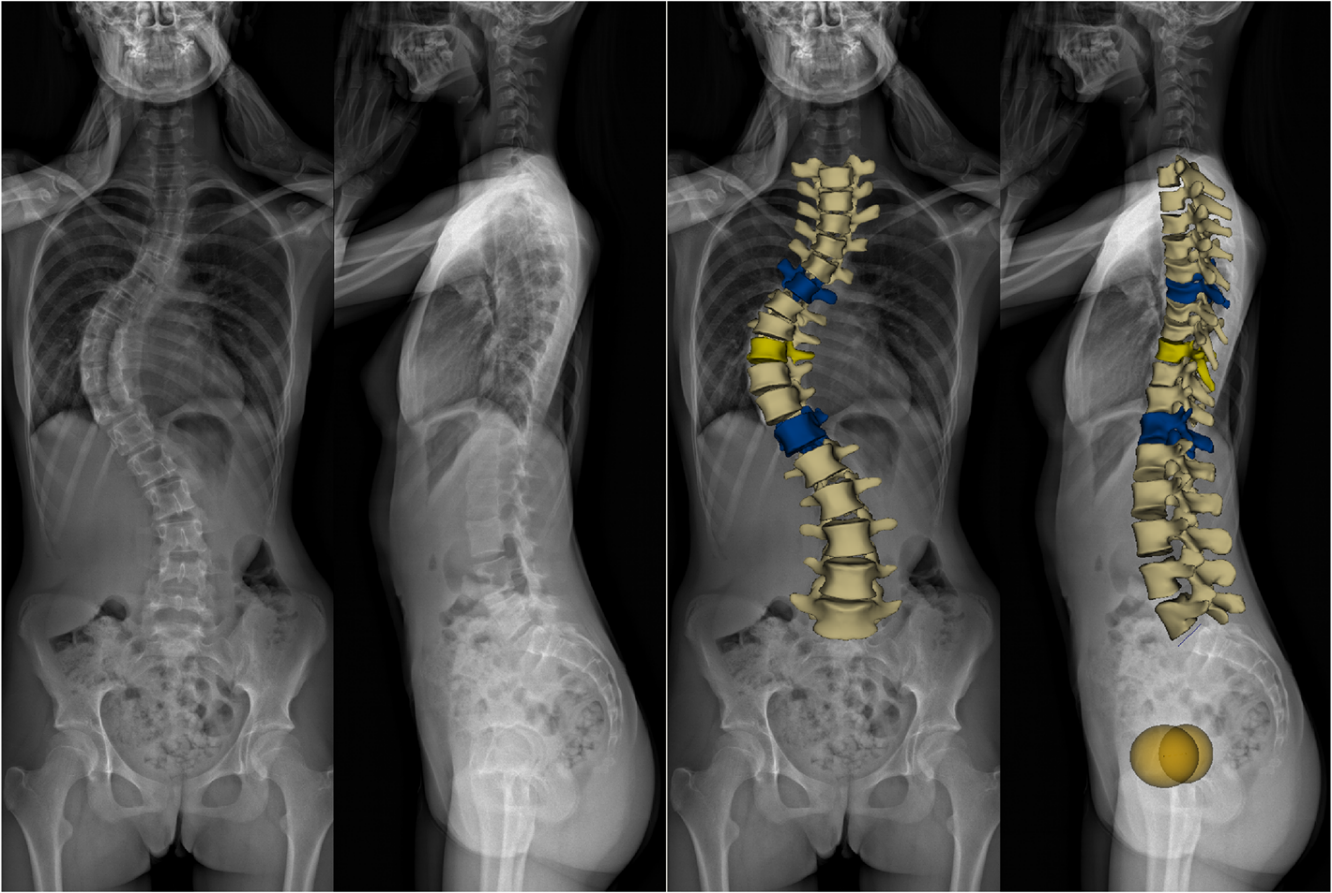
EOS 3D reconstruction. EOS scan and 3D reconstruction of a 16-year-old female patient with AIS. Cobb angle 67°; Lenke classification, 1AN

All images were recorded with the EOS 2D/3D system during routine clinical work, using the standard step-forward position defined by the EOS operating manual (right foot 5–10 cm forward, hands raised to the face with flexed elbows). After scans were collected, 3D reconstruction was performed using the sterEOS software package (v1.3.4.3740, EOS Imaging, Paris, France) (see Fig. 1). During the reconstruction process, an examiner must provide assistance to mark reference points on the images, and so, intra-observer reliability was evaluated to ensure consistency of results. The examiner reviewed 25 randomly selected cases on three separate occasions, and the intraclass correlation coefficient was calculated. Results were assessed as per the Winer criteria in which 0–0.24 is regarded as “weak or absent” reliability, 0.25–0.49 “low,” 0.50–0.69 “medium,” 0.70–0.89 “high,” and 0.90–1.0 “excellent” [24].

**Fig. 2:**
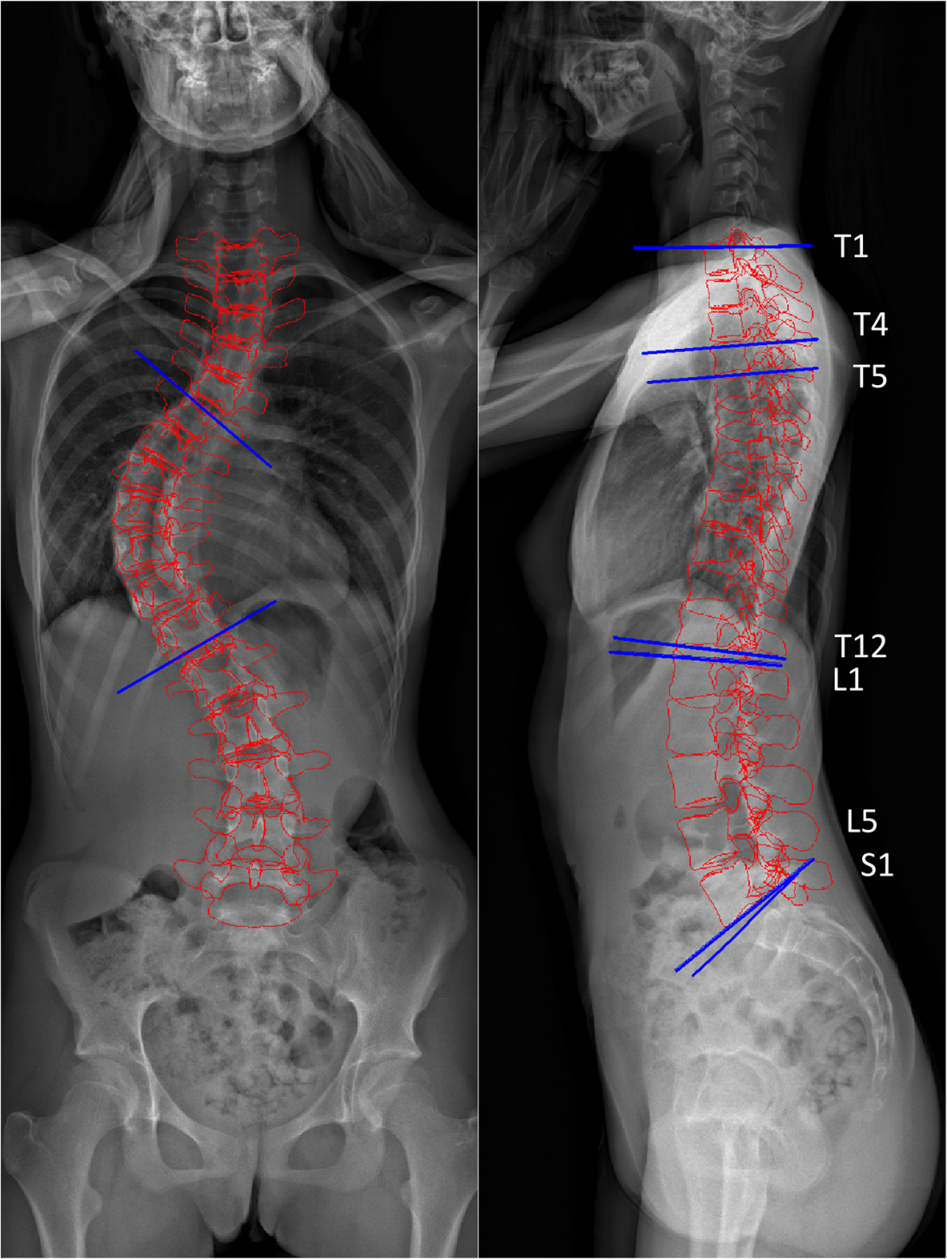
Measured spine parameters. The left picture shows the line of the superior endplate of the upper vertebra of the scoliotic curve and the line of the inferior end plate of the lower vertebra of the curve; the complementary angle of these lines is the Cobb angle The right picture shows the sagittal parameters. The kyphosis and lordosis parameters are defined as the angle between the superior endplate of the upper vertebra and inferior endplate surface of the lower vertebra

**Fig. 3:**
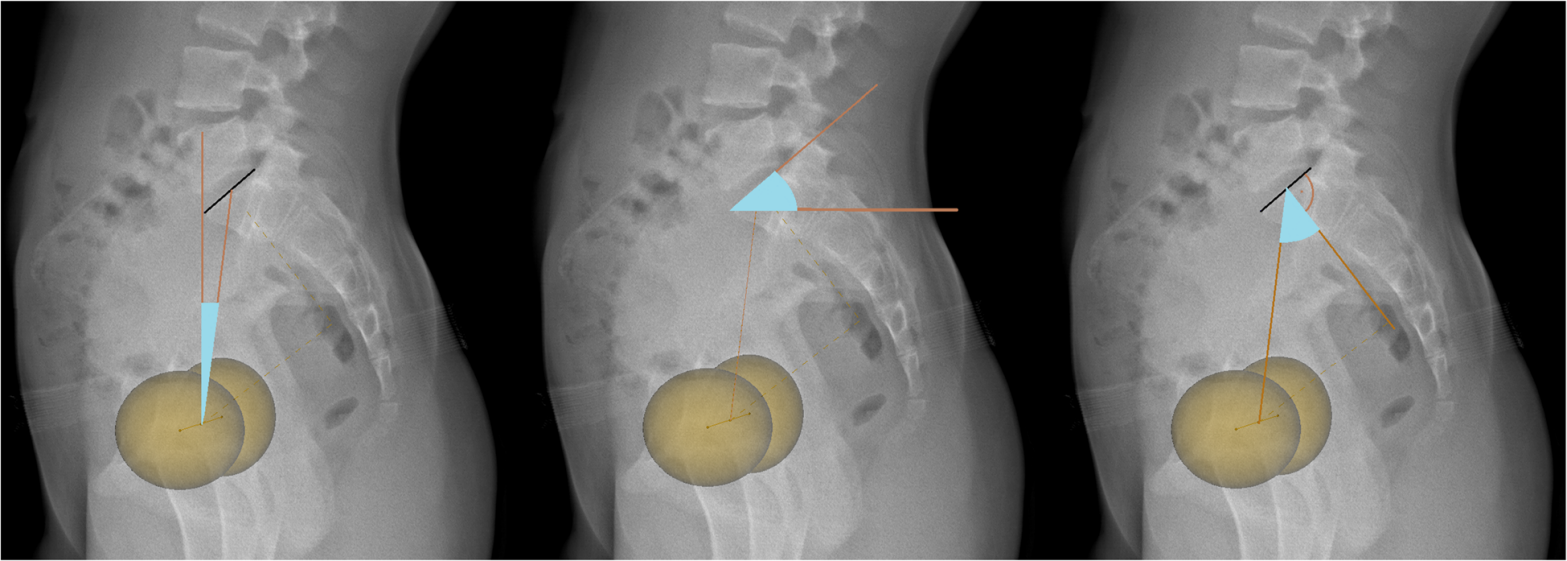
Measured pelvic parameters. From left to right: pelvic tilt, sacral slope, and pelvic incidence

The following parameters were evaluated (see Figs. 2 and 3):

- Cobb angle: the angle formed between the superior endplate of the uppermost vertebra of the scoliotic curve and the inferior endplate of the lowest vertebra of the curve;
- T1–T12 kyphosis (kyphosis and lordosis parameters are defined as the angle between the superior endplate of the upper vertebra and inferior endplate surface of the lower vertebra);
- T4–T12, T5–T12 kyphosis;
- L1–L5 and L1–S1 lordosis;
- Pelvic tilt (PT): the angle between a line running from the center of the S1 endplate to the center of the femoral head and the vertical axis (also termed the pelvic version);
- Sacral slope (SS): angle between the S1 endplate and the horizontal axis;
- Pelvic incidence: angle between a perpendicular line through the center of the first sacral vertebral endplate in the sagittal plane and a line passing from the center of the sacral plate to the center of the femoral head.

Patients were also stratified by frontal curve appearance as per the Lenke scoliosis classification [25]. Subgroups and average Cobb angles are shown in Table 1. For the Lenke subclassification, the central sacral vertical line (CSVL) and sagittal plane curvature of T2–T5 kyphosis, T5–T12 kyphosis, and T10–L2 kyphosis were also evaluated.

**Table 1:** The partition of scoliotic cases based on the Lenke classification and the average Cobb angle values of the subgroups

		L mod			S mod			Cobb angle (°)					
		A	B	C	−	N	+	Prox		MT		TL/L	
	*n =*							Mean	S.D.	Mean	S.D.	Mean	S.D.
Lenke 1	165	131	28	6	34	108	23	*–*	*–*	**36.7**	**20.4**	*–*	*–*
Lenke 2	12	6	4	2	3	5	4	**37.3**	**8.8**	**45.5**	**15.5**	*–*	*–*
Lenke 3	92	5	30	57	30	55	7	*–*	*–*	**57.9**	**22.9**	**41.0**	**15.2**
Lenke 4	8	1	3	4	3	3	2	**39.8**	**7.2**	**73.1**	**13.9**	**53.9**	**19.5**
Lenke 5	155	–	–	155	9	127	19	*–*	*–*	*–*	*–*	**26.7**	**13.1**
Lenke 6	26	–	–	26	5	20	1	*–*	*–*	**41.1**	**13.1**	**49.7**	**14.2**
Sum/average	458	143	65	250	84	318	56	**38.4**	**8.5**	**44.8**	**21.0**	**34.3**	**16.5**

*S.D.* standard deviation, *Prox* proximal curve, *MT* main thoracic curve, *L* lumbar curve, *TL* thoracolumbar curve. In Lenke 1–6, the lumbar modifier (L mod) is based on the lumbar position of the central sacral vertical line (CSVL). The sagittal modifier (S mod) is based on the value of T5–T12 kyphosis

Data in bold are significant values

The differences between the control and scoliosis groups as a whole, and by each Lenke curve type, were assessed using an independent *t* test. Linear regression was performed to evaluate the relationship between individual parameters. *p* < 0.05 was considered significant. The normalcy of distribution was assessed by the Kolmogorov–Smirnov test. All statistical analysis of the parameters was done using SPSS v22 (IBM Corp., Armonk, NY, USA) and Microsoft Office Professional Plus v14.0.6112.5000 (Microsoft Corp., Redmond, WA, USA) program packages.

## Results

Intra-observer reliabilities for 3D reconstructions were all greater than 0.9, regarded as “excellent.” The results of sagittal spine and pelvis evaluations are presented in Table 2, which shows the differences between the scoliosis cohort as a whole and the control group. Significant differences (*p* < 0.001) were seen in the thoracic region, with scoliosis patients exhibiting decreased thoracic curvatures as measured by the following: T1–T12 kyphosis (control, 43.4 ± 12.7° vs. AIS 34.1 ± 17.1°), T4–T12 kyphosis (control, 37.7 ± 15.1° vs. AIS, 27.1 ± 18.8°), and T5–T12 (control 32.9 ± 15.0° vs. AIS 24.9 ± 15.8°). No significant difference was found between the lumbar or pelvic regions of control and scoliosis patients when all scoliosis patients were averaged together, regardless of curve type (*p* values ranged from 0.290 to 0.830).

**Table 2:** Results of the sagittal parameters

	*(Degree)*	T1–T12 Kyp	T4–T12 Kyp	T5–T12 Kyp^✽^	L1–L5 Lord	L1–S1 Lord	PT	PI	SS
Control *(n = 69)*	*Mean*	43.4	37.7	32.9	46.0	57.0	7.1	46.2	39.1
	*S.D.*	12.7	15.1	15.0	9.1	10.4	7.3	8.3	6.7
AIS *(n = 458)*	*Mean*	34.1	27.1	24.9	46.4	54.9	7.5	47.3	39.6
	*S.D.*	17.1	18.8	15.8	13.2	14.8	8.3	12.8	10.3
*t* test	*p*	< 0.001	< 0.001	< 0.001	0.830	0.290	0.722	0.564	0.748

*S.D.* standard deviation, *AIS* adolescent idiopathic scoliosis, *Kyp* kyphosis, *Lord* lordosis, *PI* pelvic incidence, *PT* pelvic tilt, *SS* sacral slope

^✽^T5–T12 kyphosis measured manually on sterEOS 2D workstation

Results when patients were stratified by the Lenke curve morphology are presented in Table 3. Thoracic curvature was decreased across T1–T12, T4–T12, and T5–T12 in all groups from Lenke 1–6 compared to the control. T4–T12 kyphosis was found to be significantly lower, with different Lenke types’ mean values ranging from 18.5° to 32.6° compared to 37.7° in the control group. However, in T1–T12 kyphosis and T5–T12 kyphosis, differences were only significant in Lenke 1, 3, 5, and 6 groups (T1–T12, AIS 26.5°–38.8° vs. control 43.4°; T5–T12, 20.1°–27.7° vs. control 32.9°). In Lenke 2 and 4, group values were lowered compared to those of controls but were not significant (*p* = 0.060, 0.185). The lumbar and pelvic parameters again were not found to differ significantly from controls.

**Table 3:** The partition of sagittal parameters based on the Lenke classification

	Control	Lenke 1	C—L1	Lenke 2	C—L2	Lenke 3	C—L3	Lenke 4	C—L4	Lenke 5	C—L5	Lenke 6	C—L6
	*n = 69*	*n = 165*	*t test*	*n = 12*	*t test*	*n = 92*	*t test*	*n = 8*	*t test*	*n = 155*	*t test*	*n = 26*	*t test*
*(Degree)*	Mean ± S.D.	Mean ± S.D.	*p*	Mean ± S.D.	*p*	Mean ± S.D.	*p*	Mean ± S.D.	*p*	Mean ± S.D.	*p*	Mean ± S.D.	*p*
T1–T12 Kyp	43.4 ± 12.7	34.0 ± 17.5	< 0.001	36.9 ± 18.3	0.148	28.3 ± 18.1	< 0.001	33.4 ± 19.9	0.060	38.8 ± 14.5	0.039	26.5 ± 16.9	< 0.001
T4–T12 Kyp	37.7 ± 15.1	27.2 ± 20.9	< 0.001	27.7 ± 20.2	0.049	20.4 ± 19.2	< 0.001	23.4 ± 20.0	0.019	32.6 ± 13.8	0.022	18.5 ± 19.6	< 0.001
T5–T12 Kyp	32.9 ± 15.0	25.1 ± 18.3	0.005	26.2 ± 18.8	0.185	21.0 ± 15.9	< 0.001	23.3 ± 16.9	0.102	27.6 ± 12.5	0.012	21.1 ± 11.4	< 0.001
L1–L5 Lord	46.0 ± 9.1	46.8 ± 13.9	0.688	49.4 ± 13.6	0.285	44.5 ± 13.2	0.449	47.5 ± 8.8	0.657	47.1 ± 12.1	0.542	44.6 ± 15.9	0.630
L1–S1 Lord	57.0 ± 10.4	54.5 ± 17.7	0.310	58.3 ± 14.6	0.718	53.0 ± 12.9	0.054	55.6 ± 8.4	0.707	56.4 ± 12.7	0.724	52.9 ± 14.5	0.151
PT	7.1 ± 7.3	7.4 ± 8.0	0.821	5.2 ± 7.8	0.415	8.7 ± 9.9	0.314	6.7 ± 4.5	0.866	7.3 ± 8.1	0.862	7.0 ± 6.7	0.937
PI	46.2 ± 8.3	46.4 ± 11.9	0.934	47.5 ± 13.7	0.690	49.8 ± 14.3	0.095	49.9 ± 3.7	0.227	46.7 ± 13.1	0.822	46.3 ± 11.6	0.913
SS	39.1 ± 6.7	39.0 ± 9.8	0.926	42.3 ± 10.0	0.187	41.1 ± 10.3	0.199	43.2 ± 3.1	0.095	38.9 ± 11.4	0.896	39.5 ± 7.5	0.817

*Kyp* kyphosis, *Lord* lordosis, *PI* pelvic incidence, *PT* pelvic tilt, *SS* sacral slope, *S.D.* standard deviation. The statistical analysis of Lenke groups is compared to control with independent sample *t* test

Pelvic parameters and main sagittal curvature values were compared using linear regression analysis as seen in Table 4. The values of the lumbar lordosis showed significant correlation with PI (*p* = 0.035) and SS (*p* < 0.001) in control and in AIS (*p* < 0.001). Thoracic kyphosis showed a correlation with PT in both groups (control *p* = 0.017, AIS *p* < 0.001) and with PI in AIS (p < 0.001).

**Table 4:** The linear regression analysis

	Control						AIS					
	PI		PT		SS		PI		PT		SS	
	*B coef*	*p*	*B coef*	*p*	*B coef*	*p*	*B coef*	*P*	*B coef*	*p*	*B coef*	*p*
Thoracic kyphosis	− 0.19	0.165	− 0.32	0.017	− 0.11	0.409	− 0.20	< 0.001	− 0.21	< 0.001	0.07	0.142
Lumbar lordosis	0.28	0.035	0.26	0.058	0.63	< 0.001	0.47	< 0.001	− 0.02	0.623	0.57	< 0.001

*B coef* beta coefficient, *PT* pelvic tilt, *PI* pelvic inclination, *SS* sacral slope, *AIS* adolescent idiopathic scoliosis

## Discussion

Adolescent idiopathic scoliosis is one of the most common structural spine deformities in childhood, affecting up to 1–4% of the population [26]. Understanding of the natural history, early identification, and proper management may all be aided by high-resolution virtual visualization of the global spino-pelvic complex.

In addition to the changes seen with normal growth [16–18], the sagittal position of the pelvis is known to be altered in spino-pelvic disorders. In spondylolisthesis, for example, the pelvic incidence angle, SS, and lumbar lordosis have been found to be significantly increased, while thoracic kyphosis is decreased [14, 15]. Intervertebral disc pathology on the other hand has been associated with decreased PI values, which leads to reduced lordosis and consequently a “flatter” spine [6, 14]. The changes in scoliosis, however, are not yet clear. Some authors have described significant alterations in pelvic position [9, 10] while others did not find evidence of change [12]. Different authors have examined different ethnicities, using different parameters at various vertebral levels, however.

In the current study, we aimed to evaluate the changes of the sagittal spino-pelvic parameters in adolescent idiopathic scoliosis, in a large population of 458 Central European (Hungarian) Caucasian adolescent and young adult patients using low-distortion EOS 2D/3D reconstructions. We found no significant difference between the sagittal pelvic parameters of those with scoliosis and control individuals, even when divided by the Lenke curve type. These results were similar to those seen by other authors such as Legaye et al. or Yong et al. [12, 27].

We did not see significantly increased pelvic incidence values in scoliosis similar to those reported by Upasani et al. and Mac-Thiong et al. [9, 10]. Although PI was significantly correlated with lumbar lordosis, neither were significantly altered in our scoliosis group. Mac-Thiong et al. and Upasani et al. attributed the changes in PI to be that of a compensatory mechanism, which tries to deepen the lumbar curvature and stabilize the body’s global balance. This was especially thought to be true in the case of thoracic curves. Our results did not show a significant change in PI with thoracic curves, but there was a correlation between thoracic kyphosis and pelvic incidence, seen with linear regression analysis, that may still support this theory.

Lumbar sagittal parameters in AIS patients in our study also did not differ statistically from control values, regardless of frontal deformity appearance. This contrasts with Mac-Thiong et al., who saw a decrease of 6.7° between healthy and scoliosis children’s mean lordosis values [9, 28], but agreed with Yong et al. and their study of 95 Chinese children with AIS, who found no significant difference [27].

Values for the thoracic kyphosis however showed a large decrease in all groups when measured from T1–T12, T4–T12, and T5–T12, except for the Lenke 2 and Lenke 4 groups. This agrees with the decreased kyphosis seen in the work of Upasani et al. on Lenke curve types 1 and 5. Hu et al. and Yong et al. too found decreased thoracic kyphosis in AIS [10, 27, 29].

To our knowledge, we are presenting one of the largest populations of scoliotic individuals with spino-pelvic assessment. However, despite our large sample size, only a few clear trends were observed in our data. Even when divided by Lenke curve type, great variation was present between individuals within each type, such that group mean comparisons did not reveal significant differences in pelvic values. Inter-individual differences in pelvic and spinal parameters are known to exist in normal and scoliosis populations; however, we believe that possible ethnic differences between populations has not been paid sufficient attention. Of the publications produced by other authors that did find significant differences, not only are there distinctly different magnitudes and directions of the pelvic changes in scoliosis, there are marked differences between values for the normal populations (see Table 5). Ethnic differences in sagittal pelvic values we believe have been frequently overlooked, despite numerous publications indicating these differences.

**Table 5:** Table summarizing recent studies of interest of sagittal spino-pelvic position in different ethnicities, in normal and scoliosis populations

	Ethnicity		Type	Subjects	PI	SS	PT	L1–L5 Lord	L1–S1 Lord	Age
Current study	Caucasian		asx	69	46.2 ± 8.3	39.1 ± 6.7	7.1 ± 7.3	46.0 ± 9.1	57.0 ± 10.4	17.1 ± 4.4
			scol	458	47.3 ± 12.8	39.6 ± 10.3	7.5 ± 8.3	46.4 ± 13.2	54.9 ± 14.8	16.8 ± 4.7
Mac-Thiong et al. [28]	Caucasian (N. American)		asx	341	49.1 ± 11.0	41.4 ± 8.2	7.7 ± 8.0	48.0 ± 11.7	x	12.1 ± 3.3
Mac-Thiong et al. [30]	Caucasian (N. American)	*F*	asx	709	52.4 ± 10.8	39.8 ± 7.9	12.7 ± 7.0	x	x	36.8 ± 14.3
		*M*			52.7 ± 10.0	39.3 ± 8.0	13.4 ± 6.7			
Roussouly et al. [6]	Caucasian		asx	160	51.9 ± 10.7	39.9 ± 8.2	12.0 ± 6.5	x	61.4 ± 9.7	27 †
Mac-Thiong et al. [8]	Caucasian		scol	160	57.3 ± 13.8	47.8 ± 9.3	9.5 ± 8.7	41.3 ± 10.9	x	13.5 ± 2.0
Lonner et al. [13]	Caucasian		scol	421	52.5 †	42.2 †	10.8 †	x	59.1 †	14.8 †
	African American		scol	115	56.0 †	42.5 †	13.9 †	x	63.6 †	15.0 †
Zárate-K et al. [32]	Mexican		asx	202	56.7 ± 13.4	40.9 ± 10.6	15.8 ± 13.4		60.2 †	46.5 †
Bakouny et al. [33]	Lebanese		asx	92	52.0 ± 11.3	41.2 ± 7.9	10.8 ± 7.0	x	61.6 ± 9.2	21.5 ± 2.2
Yong et al. [27]	Chinese		asx	33	44.6 ± 11.5	33.3 ± 8.2	11.3 ± 10.8	x	49.3 ± 9.9	13.7 †
			scol	95	44.2 ± 10.0	35.1 ± 7.9	9.2 ± 8.5	x	48.5 ± 11.2	14.1 †
Zhu et al. [7]	Chinese		asx	260	44.6 ± 11.2	32.5 ± 6.5	11.2 ± 7.8	x	48.2 ± 9.6	34.3 ± 12.6
Hu et al. [29]	Chinese		scol	184	43.1 ± 10.1	37.5 ± 8.8	5.5 ± 6.9	x	55.8 ± 12.2	15.5 ± 3.3

*F* female, *M* male, *S.D.* standard deviation, *PT* pelvic tilt, *PI* pelvic inclination, *SS* sacral slope, *Lord* lordosis. Studies were included if they contained data on PI, SS, PV, and lumbar and thoracic curvatures

†Standard deviation information could be found in this paper

Reports on sagittal alignment in Chinese, Caucasian, and African-American cohorts, for example, have revealed significant differences especially in pelvis orientation. In a study by Zhu et al. of a normal Chinese population, the pelvic incidence was 44.6° (± 11.2°), and in a study by Hu et al. of a scoliotic Chinese population, pelvic incidence was 43.1° (± 10.1) [7, 29]. These values are all lower than those found in Caucasian populations, as reported by Mac-Thiong et al. and Roussouly et al. In their studies in Caucasian populations, the pelvic incidence in normal individuals was much closer to 50°, with values of 49.1° (± 11.0°), 51.9° (± 10.7°), and 52.4° (± 10.8°) (female)–52.7° (± 10.0°) (male) [6, 28, 30]. Caucasian patients with scoliosis had even higher values with mean pelvic incidence of 57.3° (± 10.9°) [9]. Interestingly, in the values reported from the Chinese population, the pelvic incidence was found to fall very slightly in those with scoliosis, in contrast to the notable increase in the Caucasian populations. Furthermore, African-Americans were reported to have higher pelvic incidences than Caucasians in cadaveric specimen and radiological studies, on average 3.5–4.1° higher, although absolute values differed from study to study [13, 31]. To us, this raises questions not only about ethnic diversity in pelvic shape in normal populations, but also about how pelvic compensatory responses to scoliosis-associated imbalances may differ due to different pelvic shapes. It must be noted, however, that marked differences between individuals were present too, though the mean group values clearly differ.

We present the data from these 458 AIS patients as a representative sample of a Caucasian Central European population, as this is the largest published radiological spino-pelvic assessment study, to our knowledge.

Recent literature has indicated that for effective treatment and planning, emphasis must be put on the correct evaluation and treatment of the sagittal condition of the spine and pelvis [3]. However, as can be seen by our results and those of other recent studies, uncertainty and controversy still exist over the assessment and definition of the normal values of the spino-pelvic complex. Due to inter-individual differences and possible ethnic differences, we still cannot confidently predict sagittal deformity from frontal images nor predict the sagittal effect of different curve types on other regions of the spine. As a result, individual assessments must be performed on all patients to ensure optimal treatment outcomes.

The main limitations of our study are the relatively low number of individuals in the control group (69 individuals) and the lower patient numbers for Lenke groups 2, 4, and 6, which may have led to a higher likelihood of observing significant differences in these cases. It must also be noted that the step-forward position with raised hands may affect the position of the pelvis and the spine. For this reason, a consistent and strict positioning protocol was applied in this study in an attempt to keep this potential effect to a minimum.

## Conclusions

This study presents the sagittal profile of 458 children with AIS, from a Central European Caucasian population, as assessed by full-body biplanar X-ray scanner. Adolescent idiopathic scoliosis in our population was connected to a significant decrease in thoracic kyphosis but did not show a significant change in pelvic alignment. This study indicates that the spino-pelvic unit sagittal alignment is not uniform. In both healthy individuals and those with spinal disorders such as scoliosis, distinct differences can be shown in different ethnic groups, in addition to inter-individual differences. In spinal deformities, the sagittal appearance cannot be deduced from frontal curvature images, and so, in all cases, an individual, personalized sagittal assessment is recommended.

## Abbreviations

2D: Two dimensional
3D: Three dimensional
AIS: Adolescent idiopathic scoliosis
B coef: Beta coefficient
CSVL: Central sacral vertical line
Kyp: Kyphosis
L mod: Lumbar modifier
L: Lumbar
Lord: Lordosis
MT: Main thoracic curve
PI: Pelvic incidence
Prox: Proximal curve
PT: Pelvic tilt
S mod: Sagittal modifier
S.D.: Standard deviation
SS: Sacral slope
TL: Thoracolumbar curve
